# State-of-art review of current malleable penile prosthesis devices in the commercial market

**DOI:** 10.1177/17562872231179008

**Published:** 2023-07-14

**Authors:** Eric Chung, Juan Wang

**Affiliations:** AndroUrology Centre, Suite 3, 530 Boundary Street, Brisbane, QLD 4000, Australia; Department of Urology, Princess Alexandra Hospital, University of Queensland, Brisbane, QLD, Australia; Macquarie University Hospital, Sydney, NSW, Australia; AndroUrology Centre, Brisbane, QLD, Australia

**Keywords:** clinical outcomes, malleable device, mechanics, patient selection, penile prosthesis

## Abstract

The malleable penile implant is often considered an inferior device to the three-piece inflatable penile prosthesis implant. Nonetheless, the malleable prosthesis has its unique advantages such as lower cost, easier to perform and fewer mechanical complications than inflatable prostheses. Furthermore, its role can be extended to patients with issues relating to poor manual hand dexterity, those undergoing a salvage for infection prosthesis and as an emergency surgical measure in patients presenting with acute ischaemic priapism. Over the past few decades, there have been numerous design and technological advancements to improve overall clinical efficacy, mechanical durability, axial rigidity and device concealability of malleable penile prostheses. The following article provides a narrative review of the six major contemporary malleable penile prosthesis devices in the commercial market, namely, the Coloplast Genesis prosthesis, the Boston Scientific Tactra prosthesis, the Zephyr ZSI 100 and 100 (female-to-male) FTM devices, the Rigi10 prosthesis, the TUBE malleable prosthesis and the Shah prosthesis and evaluates the published outcomes. Appropriate patient selection and strict counselling regarding what to expect with malleable prostheses coupled with adherence to safe surgical principles are paramount to ensure excellent clinical success and patient satisfaction rates.

## Introduction

While the introduction of the modern inflatable penile prosthesis (IPP) implant has revolutionized the treatment for erectile dysfunction (ED), the malleable penile implant was the first mainstream penile implant of the modern era since the late 1960s following the creation of a silicone prosthesis.^1^ Malleable (also known as noninflatable) prostheses are often less costly, easier to perform and have fewer mechanical complications than inflatable prostheses. It is known that key factors such as device cost, hand dexterity, state of the penile tissue and patient’s medical comorbidities will play a role in the decision-making on the type of penile prosthesis surgery.^2,3^

For malleable implant recipients, the penis remains in a semirigid state permanently, and the patient can bend the prosthesis upwards to engage in sexual intimacy. Drs Small and Carrion were largely credited for introducing modern and successful Small-Carrion malleable penile prosthetic implantation in patients.^4^ Over the next two decades, many malleable prostheses were produced and marketed and some even reach commercial success with high patient satisfaction rates such as the Jonas malleable penile prosthesis^5^ and the AMS (now owned by Boston Scientific) malleable 600 model series, Duraphase-II and Spectra malleable penile prosthesis.^6^

**Figure 1.**
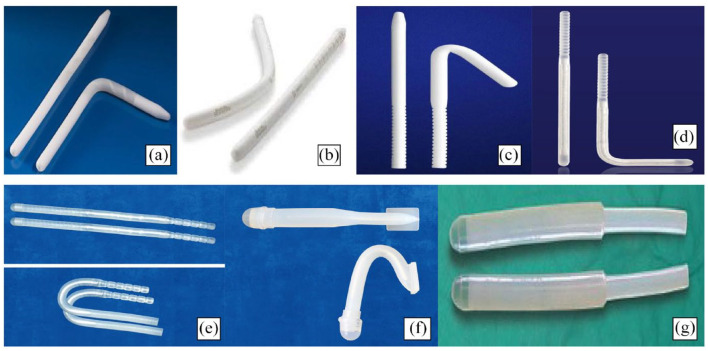
Commercially available malleable penile prosthesis devices. (a) Coloplast Genesis prosthesis. (b) Boston scientific Tactra prosthesis. (c) Rigi10^®^ prosthesis. (d) Promedon TUBE malleable prosthesis. (e) Zephyr ZSI 100 (malleable) prosthesis. (f) Zephyr ZSI 100 FTM prosthesis. (g) Shah prosthesis.

At present, there are six major malleable penile prosthesis devices available in the commercial market (Figure 1): the Coloplast Genesis prosthesis (Coloplast Corp., Minneapolis, MN, USA), the Boston Scientific Tactra prosthesis (Boston Scientific, Marlborough, MA, USA), the Zephyr ZSI 100 and 100 FTM devices (Zephyr Surgical Implants SRAL, Geneva, Switzerland), the Rigi10^®^ prosthesis (Rigicon Inc, Ronkonkoma, NY, USA), the TUBE malleable prosthesis (Promedon, Cordoba, Argentina) and the Shah prosthesis (Surgiwear, Uttar Pradesh, India). These malleable prostheses have undergone stringent internal clinical testing and external review from relevant national regulatory bodies to ensure these devices are safe and mechanically reliable. The following article provides an overview of each of these malleable prostheses in terms of device specifications and evaluates the current evidence on their clinical outcomes.

## Materials and methods

This narrative review evaluates commercially available malleable penile prostheses currently available in the market and involves a review of the literature published on MEDLINE and EMBASE databases from June 1990 to June 2022. The following key terms, namely, ‘malleable penile prosthesis’ and ‘noninflatable implant’ were utilized. Relevant published guidelines on penile prosthesis implantation were reviewed and synthesized into this review. This narrative review summarizes relevant features unique to the design and technology for each malleable prosthesis (Table 1) and published studies on these malleable devices. Ethics approval is not required as data are readily available in public domains.

**Table 1.** Currently available malleable penile prostheses in the commercial market.

Name	Company	Materials	Sizes	Device bend flexibility
Genesis	Coloplast	Flexible silicone elastomer with a hydrophilic polyvinylpyrrolidone coating	Diameter Length 13 mm 18–27 cm 11 mm 16–25 cm 9.5 mm 14–23 cm	90 degree
Tactra	Boston Scientific	Dynamic Nitinol (nickel–titanium alloy) core encased by dual-layer silicone exterior	Diameter Length 13 mm 18–27 cm 11 mm 16–25 cm 9.5 mm 14–23 cm	120 degree
Rigi10	Rigicon Inc	Flexible silicone elastomer with a HydroShield coating	Diameter Length 9 mm 23 cm 10 mm 23 cm 11 mm 25 cm 12 mm 25 cm 13 mm 25 cm 14 mm 25 cm	135 degree
Zephyr ZSI 100	Zephyr Surgical Implants	NUSIL silicone cylinder with an inner plate of Nitinol (nickel–titane) cable and a flexible distal part	Diameter Length 11 mm 12–25 cm	Unknown
TUBE malleable	Promedon	Flexible silicone elastomer with a soft distal part, medium hardness middle part and high hardness proximal part with a silver core wire	Diameter Length 9 mm 20 cm 10 mm 22 cm 11 mm 24 cm 12 mm 26 cm 13 mm 26 cm	130 degree
Shah malleable	Surgiwear	Flexible silicone elastomer consisting of four parts [a soft distal silicon tip, an anterior segment of very stiff silicon, a central 5 cm zone of soft silicon (provides flexible hinge) and a moderately firm posterior crural segment]	Diameter Length 9 mm 9–10 cm 11 mm 11–12 cm 13 mm 24 cm 15 mm 26 cm	

## Malleable prosthesis implants: clinical data

### Coloplast Genesis (Coloplast)

#### Manufacturer, commercial date and device specifications

The Coloplast Genesis was introduced in 2004.^7^ It consists of a flexible silicone elastomer device with design specifications including a hydrophilic polyvinylpyrrolidone coating that enables a surgeon to select and customize the choice of antibiotic(s) preparation and elution. The device has a silver core but no internal springs, cables or moving components to ensure mechanical reliability and prevents the device from auto spring-back. Its distal shaft column has sufficient rigidity to prevent device buckling during sexual activity.

The Genesis prosthesis rod can be trimmed to the appropriate length and available cylinder lengths were 14–23 cm, 16–25 cm and 18–27 cm while the cylinder diameter comes in sizes 9.5, 11 and 13 mm. No special tools are required, and three different sizes of tail caps (0, 0.5 and 1 cm) are available for adjusting the prosthesis length. The bend flexibility for the Genesis rod is around 90 degrees.

#### Clinical outcomes

Earlier publication on Genesis prostheses reported excellent clinical outcomes in patients with combined ED and Peyronie’s disease.^8^ In a comparative study between the Coloplast Genesis model and the older AMS Spectra device,^9^ there was no significant difference detected between the devices (77.1% *versus* 75.6%, *p* = 0.497) to indicate a difference in device superiority. Another recent publication highlighted that a larger diameter Genesis device was associated with higher complication rates without higher patient satisfaction rates.^10^

### Tactra malleable (Boston Scientific formerly American Medical Systems)

#### Manufacturer, commercial date and device specifications

The Tactra™ malleable penile prosthesis was introduced in 2019 and in comparison with the original Spectra device, this next-generation malleable prosthesis from Boston Scientific company has a dynamic Nitinol (nickel–titanium alloy) core encased by a proprietary dual-layer silicone exterior to provide device rigidity and durability.^11^

Similar to the Genesis device, the Tactra implant has trimmable exterior etchings for corporal size optimization and is available in three different cylinder lengths of 14–23 cm, 16–25 cm and 18–27 cm with corresponding cylinder diameters 9.5, 11 and 13 mm. An insertion-fit rear-tip extender is available in 0, 0.5 and 1 cm to adjust the final cylinder length. The bend flexibility for the Tactra device is 120 degrees.

#### Clinical outcomes

To date, there is no published study on the Tactra device.

### Rigi10™ (Rigicon Inc)

#### Manufacturer, commercial date and device specifications

The Rigi10™ malleable prosthesis is a relatively new device introduced in 2019.^12^ It has a propriety HydroShield coating that is hydrophilic in nature to allow for easier device implantation and choice of antibiotic elution. Like the Coloplast Genesis device, it is made of flexible silicone elastomer and has a good shape memory that minimizes spring backs. The Rigi10™ prosthesis is available in six different diameters (9, 10, 11, 12, 13 and 14 mm) and two lengths of 23 cm (for 9- and 10-mm diameters) and 25 cm (11-, 12-, 13- and 14-mm diameters). Secure-Fit Extenders come in 0.5 and 1.0 cm sizes to cap the distal end of the rod. The Rigi10™ device has a reported 135-degree flexibility.

#### Clinical outcomes

To date, there is no published study on the Rigi10 device.

### Zephyr ZSI 100 and ZSI 100 FTM malleable implant (Zephyr Surgical Implants SRAL)

#### Manufacturer, commercial date and device specifications

In contrast to the previous three malleable implants produced in the United States, the Zephyr ZSI malleable implant series is produced by a European manufacturer. The Zephyr ZSI 100 malleable implant was introduced in 2020 and is made of a Nusil silicone cylinder with a reinforced inner plate Nitinol (Nickel–Titane) cable to ensure mechanical reliability and a flexible distal part for optimum gland comfort in a flaccid position.^13,14^ The cylinder rod is available in a single size of 11 mm in diameter and 12–25 cm in length that consists of an 8-m distal part (can be cut at 5 mm each), a 12-cm central part (bendable) and a 5-cm proximal part (can be cut at 10 mm each). In contrast, the unique Zephyr ZSI 100 female-to-male (FTM) malleable implant is designed specifically for trans males, and it has an adjustable distal end from 13 to 16 cm and a cylinder width of 22 mm.^15^ It has a separate 25 mm wide, glans-shaped ‘stopper’ that can be affixed to the distal tip of the implant while the proximal part is made of silicone and stainless steel for fixation onto the pubic bone. There is no information about the bending flexibility on both Zephyr malleable rods.

#### Clinical outcomes

While there is no published data on the Zephyr ZSI 100 (normal) malleable implant, there is one study published on Zephyr ZSI 100 FTM malleable implant.^16^ While the Zephyr ZSI 100 FTM was successfully placed in 24 transgender males following phalloplasty, it was associated with high complication rates in eight patients (32%), namely, prosthetic infection (*n* = 3), protrusion (*n* = 4) or pubic pain (*n* = 1), and an additional three patients had their prostheses explanted due to difficulty living with the malleable prosthesis. Of those with the prosthesis in place, 13 of 14 patients (93%) were able to engage in penetrative sexual intercourse.

### TUBE malleable prosthesis (Promedon)

#### Manufacturer, commercial date and device specifications

The TUBE (Promedon) malleable prosthesis was marketed back in 2006.^17^ It consists of a graded silicone elastomer ranging from soft hardness at the distal tip, medium hardness in the middle section and high hardness silicone in the proximal end (for anchorage into the corporal body).^18^ The distal two-thirds of the device is term functional length and consists of a polytetrafluoroethylene (PTFE)-covered silver twisted wire core to ensure axial rigidity yet provide up to 130 degrees of malleability for the distal component. Its proximal third has multiple 5 mm circular marks for trimming to the required length. Two rear-tip extender caps are available in 10 mm and 15 mm sizes. There is no information about the bending flexibility of this TUBE malleable device.

#### Clinical outcomes

There are two studies published on Promedon TUBE prosthesis. An earlier study^19^ consisting of 83 patients reported successful sexual intercourse in 75 (90.4%) of cases and complaints of prosthesis too short were recorded in 27 (32.5%) patients. Common complications encountered include crural cross-perforation (4%), penile hematomas (1.6%) and penile hypoesthesia (0.8%). A larger study published almost a decade later by a different group^20^ on 128 patients showed an overall satisfaction rate of 78.5% with relatively low complication rates such as glans urethral injury (1.5%), acute urinary retention (3.9%), superficial wound infection (7%), penile discomfort (9.4%) and penile prostheses infection (5.5%).

### Shah malleable prosthesis (Surgiwear)

#### Manufacturer, commercial date and device specifications

The first prototype of the Indian malleable penile implant was manufactured in 1996, and over the years, the Shah Indian penile prosthesis has evolved through many models.^21^ The latest Shah device consists of a silicon elastomer and has four parts, namely, a soft distal silicon tip, an anterior segment of very stiff silicon (for penile rigidity), a central 5 cm zone of soft silicon (acts as a flexible hinge at the base of the penis for concealment) and a moderately firm posterior crural segment that could be trimmed and fitted with rear-tip extenders.^22^ The Shah Penile Prosthesis is available in two models, namely, (1) with a hinge and (2) without a hinge. Both types and all sizes of prostheses are covered with two removable outer sleeves of silicone elastomer, which can be removed to adjust the diameter of the prosthesis (this minimizes the inventory).

Shah prosthesis with hinge has a flexible central area that acts as a hinge to allow the penis to be bent for concealment when not in use. The distal one-third is made of firm silicone while the middle segment is made of soft silicone to provide a hinge effect, and the proximal one-third is made of medium-hard silicone (this portion can be cut to adjust the total length of the implant).^21^ On the contrary, the Shah prosthesis without hinge is a semi-rigid implant with uniform stiffness that is adequate for intercourse while being flexible enough to permit concealment. It can be trimmed from the proximal end so that a single implant can be adjusted to any penile length.^22^ This device is available in two grades of firmness (hardness), namely, medium firm implant (known as a regular prosthesis) while the softer one (called a soft prosthesis) is often reserved for high-risk patients or difficult cases (to minimize the risk of extrusion).^21^

#### Clinical outcomes

In contrast to the last five malleable devices, the sale of the Indian malleable penile implant is largely confined to the local Indian market only and costs considerably less compared with ‘Western’ malleable prostheses.^23^ To date, three reports have been published with the Shah penile prosthesis; a single case report in a neophallus^24^ while the other study showed good residual penile tumescence in 50% of cases and more than 90% of patients reported no problem with device concealment.^25^ In another paper comparing Shah’s device and AMS 650 prostheses, there were no significant differences reported in the ED Inventory of Treatment Satisfaction (EDITS) questionnaire and EDITS partner survey at 80.66 ± 4.49 and 75.66 ± 6.57, respectively, at 12 months after surgery and 71.73 ± 8.10 and 65.6 ± 6.49, respectively, at 24 months after surgery.^26^ Major and minor postoperative complications were seen in 10.7% (one infection, one urethral injury and one impending erosion) and 21.4% (6/28) of patients, respectively.

## Conclusion

Over the past few decades, there have been numerous design and technological advancements to improve overall clinical efficacy, mechanical durability, axial rigidity and device concealability of malleable penile prostheses. While a malleable prosthesis will never match the ‘naturalness’ of an IPP, there are still certain situations and conditions in which the simplicity of a rod may be preferred over an inflatable device. A pair of malleable rods have been shown to have less risk of malfunction and need for revision surgery. Furthermore, a malleable prosthesis is significantly cheaper than an IPP, and this could play an important factor if the patient is not privately insured and would need to pay for the device. The implantation of a malleable prosthesis is easier and faster with minimal intraoperative device preparation. In addition, its role can be extended to patients with poor manual hand dexterity, those undergoing a salvage for infection prosthesis and as an emergency surgical measure in patients presenting with acute ischaemic priapism.^27–30^ Finally, in patients compromised by infection or priapism, the rods can be successfully exchanged for an IPP later, with potentially longer and wider cylinders for a greater patient satisfaction rate.^31^

Future research and development into the malleable device are likely to reside in more advanced materials such as the Nitinol exoskeleton^32^ with better axial rigidity, girth and bending flexibility for concealment. At this stage, there is no direct comparative study among these malleable prostheses in terms of clinical efficacy, mechanical durability or safety outcomes. Proper patient selection and strict counselling regarding what to expect with malleable prosthesis coupled with adherence to safe surgical principles are paramount to ensure excellent clinical success and patient satisfaction rates.

## Declarations

**Ethics approval and consent to participate:** Not applicable.

**Consent for publication:** Not applicable.

**Availability of data and materials:** Not applicable.

## References

[1] Chung E. Penile prosthesis implant: scientific advances and technological innovations over the last four decades. Transl Androl Urol 2017; 6: 37–45. 28217449 10.21037/tau.2016.12.06 PMC5313299

[2] Levine LA Becher EF Bella AJ , et al. Penile prosthesis surgery: current recommendations from the International Consultation on Sexual Medicine. J Sex Med 2016; 13: 489–518. 27045255 10.1016/j.jsxm.2016.01.017

[3] Scherzer ND Dick B Gabrielson AT , et al. Penile prosthesis complications: planning, prevention, and decision making. Sex Med Rev 2019; 7: 349–359. 30033128 10.1016/j.sxmr.2018.04.002

[4] Martinez DR Terlecki R Brant WO. The evolution and utility of the small-carrion prosthesis, its impact, and progression to the modern-day malleable penile prosthesis. J Sex Med 2015; 12(Suppl. 7): 423–430. 26565570 10.1111/jsm.13014

[5] Montague DK. Experience with Jonas malleable penile prosthesis. Urology 1984; 23(Suppl.): 83–85. 10.1016/0090-4295(84)90248-6 6719686

[6] Dorflinger T Bruskewitz R. AMS malleable penile prosthesis. Urology 1986; 28: 480–485. 3787920 10.1016/0090-4295(86)90147-0

[7] Genesis^®^ malleable penile prosthesis, https://products.coloplast.us/coloplast/implantable-devices/mens-health-mh/erectile-dysfunction/genesis-malleable-penile-prosthesis/ (accessed 1 December 2022).

[8] Yavuz U Ciftci S Ustuner M , et al. Surgical treatment of erectile dysfunction and Peyronie’s disease using malleable prosthesis. Urol J 2015; 12: 2428–2433. 26706740

[9] Casabé AR Sarotto N Gutierrez C , et al. Satisfaction assessment with malleable prosthetic implant of Spectra (AMS) and Genesis (Coloplast) models. Int J Impot Res 2016; 28: 228–233. 27557609 10.1038/ijir.2016.33

[10] Habous M Omar M Farag M , et al. Malleable penile implant rod diameter predicts complications and patient satisfaction. Sex Med 2022; 10: 100486. 35217441 10.1016/j.esxm.2021.100486 PMC9023239

[11] Tactra™ malleable penile prosthesis, https://www.bostonscientific.com/en-US/products/penile-prosthesis/tactra—malleable-penile-prosthesis.html (accessed 1 December 2022).

[12] Rigi10™ malleable penile prosthesis, https://www.rigicon.com/malleable-penile-prosthesis/ (accessed 1 December 2022).

[13] ZSI 100 malleable penile implant, https://www.zsimplants.ch/documents/zsi100/100_Flyer-2016_V4.pdf (accessed 1 December 2022).

[14] ZSI 100 malleable penile implant, https://www.zsimplants.ch/en/products-en/erectile-dysfunction/zsi-100-std-malleable-penile-implant/zsi-100-std-information/zsi-100-standard-special-features (accessed 1 December 2022).

[15] ZSI 100 FTM malleable penile implant, https://www.zsimplants.ch/en/products-en/phalloplasty/zsi-100-ftm-malleable-penile-implant/zsi-100-ftm-information (accessed 1 December 2022). 10.1016/j.jsxm.2019.09.019 31680006

[16] Pigot GLS Sigurjónsson H Ronkes B , et al. Surgical experience and outcomes of implantation of the ZSI 100 FtM malleable penile implant in transgender men after phalloplasty. J Sex Med 2020; 17: 152–158. 31680006 10.1016/j.jsxm.2019.09.019

[17] TUBE malleable penile prosthesis, https://promedon-upf.com/product/tube/ (accessed 1 December 2022).

[18] TUBE malleable penile prosthesis, https://promedon-upf.com/wp-content/uploads/TUBE_Brochure_ENG-V5.pdf (accessed 1 December 2022).

[19] Fathy A Shamloul R AbdelRahim A , et al. Experience with tube (Promedon) malleable penile implant. Urol Int 2007; 79: 244–247. 17940357 10.1159/000107957

[20] Mohamed ER Hammady AR Eldahshoury MZ , et al. Surgical outcomes and complications of tube (Promedon) malleable penile prostheses in diabetic versus non-diabetic patients with erectile dysfunction. Arab J Urol 2016; 14: 305–311. 27900222 10.1016/j.aju.2016.07.002 PMC5122751

[21] Surgiwear, http://www.surgiwear.co.in/andrology/implants/shah-penile-prosthesis.html (accessed 1 December 2022).

[22] http://www.surgiwear.co.in/urosurgery/implants/shah-penile-prosthesis.html (accessed 1 December 2022).

[23] Shah R. Twenty-five years of the low-cost, noninflatable, Shah Indian penile prosthesis: the history of its evolution. Indian J Urol 2021; 37: 113–115. 34103792 10.4103/iju.IJU_60_21 PMC8173931

[24] Patwardhan SK Shah R Kulkarni V , et al. Shah’s Indian penile prosthesis placement after phallic reconstruction with radial forearm flap. Indian J Urol 2008; 24: 107–108. 19468370 10.4103/0970-1591.38613 PMC2684244

[25] Krishnappa P Tripathi A Shah R. Surgical outcomes and patient satisfaction with the low-cost, semi-rigid Shah penile prosthesis: a boon to the developing countries. Sex Med 2021; 9: 100399. 34274823 10.1016/j.esxm.2021.100399 PMC8360909

[26] Patil AY Nerli RB Dixit NS , et al. Satisfaction with the semirigid penile prosthesis among couples from a semiurban Indian population. J Sci Soc 2018; 45: 26–29.

[27] Köhler TS Modder JK Dupree JM , et al. Malleable implant substitution for the management of penile prosthesis pump erosion: a pilot study. J Sex Med 2009; 6: 1474–1478. 19453933 10.1111/j.1743-6109.2009.01236.x

[28] Tausch TJ Zhao LC Morey AF , et al. Malleable penile prosthesis is a cost-effective treatment for refractory ischemic priapism. J Sex Med 2015; 12: 824–826. 25536880 10.1111/jsm.12803

[29] Chung E Gross MS Van Renterghem K , et al. Expert roundtable discussion on penile prosthesis infection prevention measures. SIU J 2021; 2: 380–381.

[30] Zacharakis E De Luca F Raheem AA , et al. Early insertion of a malleable penile prosthesis in ischaemic priapism allows later upsizing of the cylinders. Scand J Urol 2015; 49: 468–471. 26116193 10.3109/21681805.2015.1059359

[31] Gross MS Phillips EA Balen A , et al. The malleable implant salvage technique: infection outcomes after Mulcahy salvage procedure and replacement of infected inflatable penile prosthesis with malleable prosthesis. J Urol 2016; 195: 694–697. 26343986 10.1016/j.juro.2015.08.091

[32] Le BV McVary KT McKenna K , et al. Use of magnetic induction to activate a ‘Touchless’ shape memory alloy implantable penile prosthesis. J Sex Med 2019; 16: 596–601. 30935471 10.1016/j.jsxm.2019.01.318

